# Osteopontin Is Upregulated in Human and Murine Acute Schistosomiasis Mansoni

**DOI:** 10.1371/journal.pntd.0005057

**Published:** 2016-10-18

**Authors:** Thiago Almeida Pereira, Wing-Kin Syn, Frederico Figueiredo Amâncio, Pedro Henrique Diniz Cunha, Julia Fonseca Morais Caporali, Guilherme Vaz de Melo Trindade, Elisângela Trindade Santos, Márcia Maria Souza, Zilton Araújo Andrade, Rafal P Witek, William Evan Secor, Fausto Edmundo Lima Pereira, José Roberto Lambertucci, Anna Mae Diehl

**Affiliations:** 1 Division of Gastroenterology, Department of Medicine, Duke University Medical Center, Durham, NC, United States of America; 2 Departamento de Clínica Médica, Laboratório de Doenças Infecciosas e Parasitárias, Faculdade de Medicina, Universidade Federal de Minas Gerais, Belo Horizonte, MG, Brazil; 3 Laboratório de Patologia Experimental, Centro de Pesquisas Gonçalo Moniz, Fundação Oswaldo Cruz, Salvador, BA, Brazil; 4 Immunopathogesis Section, Laboratory of Parasitic Diseases, National Institute of Allergy and Infectious Diseases, National Institutes of Health, Bethesda,MD, United States of America; 5 Section of Gastroenterology, Ralph H Johnson Veteran Affairs Medical Center, Charleston, South Carolina, United States of America; 6 Division of Gastroenterology and Hepatology, Medical University of South Carolina, Charleston, South Carolina, United States of America; 7 Liver Regeneration and Repair Research Group, Institute of Hepatology, Foundation for Liver Research, London, United Kingdom; 8 Department of Physiology, School of Medicine, National University of Ireland, Galway, Ireland; 9 Thermo Fisher Scientific, Frederick, MD, United States of America; 10 Centers for Disease Control and Prevention, Atlanta, GA, United States of America; 11 Núcleo de Doenças Infecciosas, Universidade Federal do Espírito Santo, Vitória, ES, Brazil; George Washington University, UNITED STATES

## Abstract

**Background:**

Symptomatic acute schistosomiasis mansoni is a systemic hypersensitivity reaction against the migrating schistosomula and mature eggs after a primary infection. The mechanisms involved in the pathogenesis of acute schistosomiasis are not fully elucidated. Osteopontin has been implicated in granulomatous reactions and in acute hepatic injury. Our aims were to evaluate if osteopontin plays a role in acute *Schistosoma mansoni* infection in both human and experimentally infected mice and if circulating OPN levels could be a novel biomarker of this infection.

**Methodology/Principal Findings:**

Serum/plasma osteopontin levels were measured by ELISA in patients with acute (n = 28), hepatointestinal (n = 26), hepatosplenic (n = 39) schistosomiasis and in uninfected controls (n = 21). Liver osteopontin was assessed by immunohistochemistry in needle biopsies of 5 patients. Sera and hepatic osteopontin were quantified in the murine model of schistosomiasis mansoni during acute (7 and 8 weeks post infection, n = 10) and chronic (30 weeks post infection, n = 8) phase. Circulating osteopontin levels are increased in patients with acute schistosomiasis (p = 0.0001). The highest levels of OPN were observed during the peak of clinical symptoms (7–11 weeks post infection), returning to baseline level once the granulomas were modulated (>12 weeks post infection). The plasma levels in acute schistosomiasis were even higher than in hepatosplenic patients. The murine model mirrored the human disease. Macrophages were the major source of OPN in human and murine acute schistosomiasis, while the ductular reaction maintains OPN production in hepatosplenic disease. Soluble egg antigens from *S*. *mansoni* induced OPN expression in primary human kupffer cells.

**Conclusions/Significance:**

*S*. *mansoni* egg antigens induce the production of OPN by macrophages in the necrotic-exudative granulomas characteristic of acute schistosomiasis mansoni. Circulating OPN levels are upregulated in human and murine acute schistosomiasis and could be a non-invasive biomarker of this form of disease.

## Author Summary

Schistosomiasis is a major health problem that affects over 200 million people. Symptomatic acute schistosomiasis is a systemic reaction to the worms and eggs in individuals from non-endemic areas after a primary infection. Tourists, military personnel and people who practice water sports are at risk. Although most cases resolve 90 days post infection, severe cases with massive distribution of eggs can be fatal. It is frequently misdiagnosed, under diagnosed or has delayed diagnosis because the signs and symptoms are nonspecific and eggs are usually present in stool only 6 weeks post-infection. The mechanisms underlying the pathogenesis of acute schistosomiasis are not fully elucidated and currently there is a lack of noninvasive biomarkers to diagnose this form of disease. We report that serum osteopontin levels are increased in patients with acute schistosomiasis and parallel the clinical symptoms, returning to baseline level once the granulomas were modulated and the symptoms resolve. Soluble egg antigens provoke macrophages to produce osteopontin, recruiting more macrophages to the site of injury and inducing the granulomatous reaction. This observation suggests that osteopontin plays an important role in acute schistosomiasis mansoni and could be a novel non-invasive biomarker for this form of the disease.

## Introduction

Schistosomiasis is a severe tropical disease caused by *Schistosoma spp*. flatworms that affects over 200 million of people from 76 countries and territories [1]. *S*. *mansoni* is the only species in the Americas and Brazil holds the majority of infected individuals with 25 million living in endemic areas and 4–6 million infected [2].

Infected individuals have various clinical manifestations that generally cluster into three distinct forms of the disease: acute, hepatointestinal and hepatosplenic schistosomiasis [2–5]. In patients from endemic areas, the acute phase of schistosomiasis is rarely symptomatic (0.3%) due to infection early in life (3–4 years-old) and exposure to schistosoma antigens/antibodies against antigens in-utero and/or in breast milk [3]. The majority of chronically infected patients from endemic areas (90–96%) develop the hepatointestinal form of the disease, which is asymtomatic or oligosymptomatic in most cases and characterized by granulomatous inflammation in the liver and intestines, little or no hepatosplenomegaly, and minimal liver fibrosis without any sign of portal hypertension [2, 4–7]. A small proportion (4–10%) of infected individuals from endemic areas develops the hepatosplenic form of disease characterized by hepatosplenomegaly, severe liver fibrosis and portal hypertension [2, 4–7].

Among individuals from non-endemic areas, the acute form of schistosomiasis mansoni is a systemic hypersensitivity reaction against the migrating schistosomula (pre-postural phase of infection) and mature eggs (post-postural phase of infection). This typically develops within 16–90 days after a primary infection [2]. The burden of infection (and probably host genetic background) dictates the severity of the clinical manifestations: more worm couples produce more eggs and consequently, trigger an exacerbated host immune response [2, 8].

The pre-postural phase occurs during the initial 35 days after infection and is caused by immune modifications induced by the schistosomules, immature and adult worms before laying eggs [2]. Cercarial dermatitis may occur soon after infection, but symptoms are more evident when schistosomules/immature worms arrive/grow/mature in the hepatoportal veins (peak 15–21 days post infection) [2]. High fever (38–39°C), cough, abdominal pain, discrete hepatosplenomegaly and nonspecific symptoms such as muscular pain, arthralgia and headache, are observed [2, 9]. Blood eosinophilia (10–75% of eosinophils) is frequent [2, 9]. Liver biopsy reveals discrete inflammatory infiltrate consisting of lymphocytes, eosinophils, neutrophils and macrophages surrounding schistosomules/immature worms, non-specific portal hepatitis, and sparse focal intralobular necrosis [2]. During this phase a Th1 response is predominant and an increase in pro-inflammatory cytokines such as IL-2, gamma Interferon and TNF alpha is frequently observed [2, 3]. Other less frequent clinical manifestations may be present: transverse myelitis or pseudotumoral lesions in encephalon (neural schistosomiasis) [2, 9].

The post-postural phase is initiated by egg laying (approximately 35 days post-infection) and egg maturation (which begins about 6 days later) [2]. Symptoms are aggravated, episodes of diarrhea increase, and the patient experience severe weight loss [2, 4, 8]. Clinical symptoms can continue until 90 days after infection [2, 4, 8]. Severe, toxemic forms of acute disease in which there is massive dissemination of eggs throughout the intestines and lungs may be fatal [2]. Moderate to mild disease spontaneously resolves two to three months after infection [2].

During acute schistosomiasis intense miliary distribution of eggs occurs in the liver, colons, small intestines, visceral peritoneum, abdominal lymph nodes, pancreas and lungs [2]. Periovular granulomas localize on the serosal surface of affected organs and macroscopically appear as translucent granule or nodules [2]. Microscopically, the granulomas are large (over 100 times the size of the egg), necrotic-exudative, and enriched with eosinophils [2], due to the naïve hosts’ hyperergic reaction to novel parasite antigens. In the liver, granulomas are irregularly distributed through the parenchyma and portal tracts and non-specific inflammatory cells frequently surround portal tracts. Because hepatocellular lesions are relatively mild (loss of basophilia, hydropic degeneration and rare focal necrosis), the serum aminotransferases are usually normal or slightly elevated [2, 9]. An important feature of acute schistosomiasisis is that all the granulomas are uniformly in the same necrotic-exudative phase of formation, with prominent central necrosis [2]. This finding in liver biopsies is pathognomonic of acute infection.

With egg-laying the Th2 immune response starts to suppress the initial Th1 response and IL4, IL5, IL10 and IL13 are the most predominant cytokines [2, 3]. The hyperergic, massive granulomas are modulated as the infection evolves to the chronic phase. By around 90 days post-infection, liver granulomas are smaller [2, 3, 10–12] and progressively heal by fibrosis [2, 7, 10, 12]. The symptoms usually disappear due to the modulation of the immune response to the eggs [2]. Because the signs and symptoms of acute schistosomiasis are nonspecific and diagnosis is established by presence of eggs in stools that occurs only six weeks after infection, acute schistosomiasis mansoni is frequently misdiagnosed, under diagnosed or has delayed diagnosis [2, 9]. Efforts to develop tests for earlier diagnosis of the disease have been challenging. Unfortunately, lesions similar to those observed in pre-postural phase of human acute schistosomiasis are not observed in mouse models of schistosomiasis mansoni [11, 13, 14], likely because the granulomas that form in mice are generally less necrotic than those that occur in acutely infected humans [14].

Osteopontin (OPN), a pro-inflammatory cytokine and pro-fibrogenic molecule [15–17], was recently associated with hepatosplenic schistosomiasis mansoni [18]. Soluble egg antigens (SEA) directly induce liver cells to produce OPN. Moreover, serum and hepatic osteopontin levels correlate with the degree of liver fibrosis and the level of portal hypertension, suggesting that this molecule could be a novel biomarker for hepatosplenic schistosomiais mansoni [18]. The authors observed that macrophages, stellate cells and bile ductular cells in/around the granulomatous reaction are the major sources of OPN in schistosomiasis [18]. Osteopontin was also demonstrated to play a role in recruitment and activation of macrophages/Kupffer cells, neutrophils and lymphocytes [15–17, 19]. OPN-/- mice injected with *S*. *mansoni* eggs develop abnormal granuloma formation in the lung due to reduced macrophage accumulation [20]. Since in acute schistosomiasis the liver is enriched with necrotic-exudative granulomas and there is an exacerbated immune response, our aims were to evaluate if OPN increases in acute *Schistosoma mansoni* infection of both humans and mice, and to determine if circulating OPN levels might be a novel biomarker of this infection.

## Material and Methods

### Patients

This was a comparative cross-sectional study. A total of 28 patients with acute schistosomiasis mansoni diagnosed at Tropical Diseases Outpatient Clinic of the University Hospital of Universidade Federal de Minas Gerais (Belo Horizonte, Brazil) from January 2014 to December 2015 were included in the study. Serum samples from acute patients (n = 28; age 19.8±11.8 years; 21 males/7 females) and uninfected controls (n = 21; age 27.86±9.45 years; 14 males/7 females) were collected for analysis. Formalin-fixed, paraffin-embedded liver needle biopsies were available in a subgroup of patients (n = 5). Plasma samples from uninfected controls (n = 21) and from patients with Hepatointestinal (n = 27; age 35.66±12.09 years; 16 males/7 females), Hepatosplenic (n = 39; age 38.25±9.4 years; 30 males/9 females) and Acute (n = 3; age 39±25.23; 3 males) schistosomiasis were also included in the analysis.

Diagnosis of acute schistosomiasis was based on epidemiological data (recent contact with stream water in an endemic area), clinical data (cercarial dermatitis, acute enterocolitis, fever, cough, malaise, paraplegia, pulmonary involvement, hepatomegaly and or splenomegaly), laboratory assays (eosinophilia, IgG antibodies against SWAP, *S*. *mansoni* eggs in stools or rectal biopsy fragments), and imaging techniques (Ultrasound to observe liver, spleen and intra abdominal lymph node enlargement; MRI to demonstrate spinal cord injury). To be considered as having acute schistosomiasis in the present study the participants had to have more than 1 or more symptoms/signs described above, evidence of infection (parasitologic or serologic) and reported contact with contaminated waters. All patients included in the study were residents of the metropolitan region of Belo Horizonte (capital of Minas Gerais state), a non-endemic area for schistosomiasis mansoni. No previous history of contact with *S*. *mansoni* was reported by the patients or parents/guardians.

The present study was conducted in accordance with the Declaration of Helsinki (2013) of the World Medical Association and was approved by the Ethics Committee of Universidade Federal de Minas Gerais, Belo Horizonte, Minas Gerais, Brazil (UFMG) (Protocol ETIC 204/06). Written informed consent was obtained from all participating subjects or their parents/guardians (on behalf of child participant). All data regarding human participants was anonymized.

### Animal Studies

Female Swiss Webster outbred mice were infected with 50 cercariae of *S*. *mansoni* (Feira de Santana strain, CPqGM/FIOCRUZ) for 6, 7, 8 weeks (acute phase, n = 15) and 30 weeks (chronic phase, severe fibrosis, n = 8). Uninfected, age- and strain-matched animals were used as controls (n = 8). Liver tissue and serum were collected for analysis. The present study protocol meet the regulation and guidelines of Brazil’s National Animal Experimentation Control Board (CONCEA) and was approved (Protocol 003/2010) by the Ethical Committee for Animal Research of Centro de Pesquisas Gonçalo Moniz, Oswaldo Cruz Foundation, Salvador, Bahia, Brazil (CPqGM/FIOCRUZ).

### Osteopontin ELISA

OPN was quantified in the serum (humans and mice) or plasma (humans) using OPN Quantikine ELISA kit (R&D Systems) according to the manufacturer’s protocol.

### Immunohistochemistry

Liver sections were stained with H&E (haematoxylin and eosin) for general histology. Immunohistochemistry (IHC) analysis was performed to evaluate the expression of osteopontin (R&D Systems; Antigen retrieval: 3% pepsin digestion for 10 min at 37°C; 5ug/mL of primary antibody, incubation overnight at 4°C). To confirm that Macrophages produce osteopontin, double IHC was performed using the chromagen DAB (3,3_-diaminobenzidine) for OPN and the chromagen Vina Green for CD68 (a macrophage marker).

OPN staining was quantified in 15 x200 fields/sample by computer-assisted morphometry using MetaMorph (Universal Imaging Corp.). OPN (+) bile ducts were counted in 15 x200 fields/sample by three independent observers.

### SEA preparation and stimulation of primary human Kupffer cells

The SEA was prepared at Centers for Disease Control and Prevention (CDC) as previously described [21]. The amount of Gram-negative bacterial endotoxin present in the SEA preparation was quantified using the end-point chromogenic limulus amebocyte lysate assay (Lonza). To investigate if macrophages produce osteopontin, primary human Kupffer cells (from Thermo Fisher Scientific) were incubated with 10 μg/ml SEA or 0.0001 μg/ml LPS (lipopolysaccharide; control, same amount of endotoxin present in the SEA preparation) for 3,6,12 and 24 hours. RNA was collected for analysis.

### RNA analysis

RNA was extracted using RNeasy mini kit (Qiagen) according to the manufacture’s protocol. Reverse transcription was performed using the First Strand Superscript III kit (Life Technologies) using the random hexamers protocol. Osteopontin mRNA expression was evaluated by real-time PCR (Taqman, Thermo Fisher Scientific). Each sample was analysed in duplicate and target gene levels in treated cells are shown as a ratio to levels detected in corresponding control samples, according to the ΔCT method, relative to the housekeeping gene (18s). The probes were designed by Thermo Fisher Scientific.

### Statistical Analysis

Results are expressed as means ± S.E.M. (Standard Error of the Mean; for normal distribution variables) or as medians (for non-normal distribution variables). Comparisons between groups were performed using the oneway ANOVA and Student’s t test (parametric) or Kruskal–Wallis one-way ANOVA and Mann–Whitney U test (non-parametric). Significance was accepted at the 0.05 level; Bonferroni correction was applied when comparing more than two groups. Receiver operating characteristics (ROC) curve analysis was used to investigate if sera OPN levels could be a good biomarker for symptomatic acute schistosomiasis. All statistical analyses were performed using SPSS Statistics 22 (IBM) and Prism 6 (GraphPad).

## Results

Our cohort of patients consisted of classic cases of symptomatic acute schistosomiasis mansoni, fulfilling the criteria for case definition of the acute form of the disease. The most frequent symptoms in acute cases are depicted in Table 1.

**Table 1:** Main symptoms and blood eosinophils in patients with acute schistosomiasis mansoni diagnosed at Tropical Diseases Outpatient Clinic of the University Hospital of Universidade Federal de Minas Gerais (Belo Horizonte, Brazil) from January 2014 to December 2015 and included in the study.

Main symptoms^*^	N (%)
Fever	21 (91.3)
Diarrhea	14 (60.9)
Headache	13 (56.5)
Abdominal pain	12 (52.2)
Hepatomegaly	9 (39.1)
Cough	7 (30.4)
Unspecific (muscle pain, fatigue or hyporexia)	15 (65.2)
Nausea and vomiting	6 (26.1)
Weight loss	4(17.4)
Esplenomegaly	3(13.0)
Cercarial dermatitis	3(13.0)
	**Median (lowest-highest value)**
**Blood eosinophilia (%)**^**#**^	24 (13.2–78)

*data from 23 patients; Three patients presented severe colitis; one patient presented myeloradiculopathy and one developed a severe pulmonary form.

^#^data from 16 patients.

### Serum and hepatic osteopontin is increased in human acute schistosomiasis

**Fig 1:**
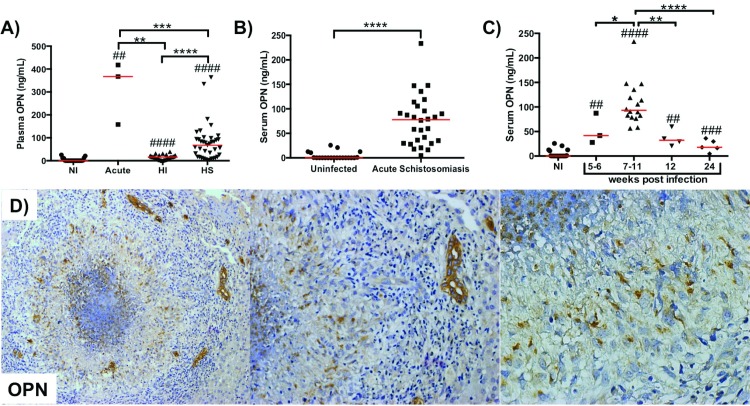
Sera and hepatic osteopontin are upregulated in human acute schistosomiasis mansoni. A) ELISA for osteopontin in the plasma of uninfected controls (NI, n = 21) and schistosomiasis patients with Acute (n = 3), hepatointestinal (n = 26) and hapatosplenic (n = 39) forms of disease. B) ELISA for osteopontin in the serum of uninfected controls (n = 21) and patients with acute schistosomiasis mansoni (n = 28). C) ELISA for osteopontin in the serum of uninfected controls (NI, n = 21) and patients with acute schistosomiasis mansoni (n = 28) grouped by weeks post infection. Medians are displayed; Mann-Whitney U test: #p<0.05, ##p<0.01, ###p<0.001, ####p<0.0001 vs uninfected controls; *p<0.05, **p<0.01, ***p<0.001, ****p<0.0001. D) Immunohistochemistry for osteopontin in liver needle biopsy fragment of a representative subject with acute schistosomiasis mansoni (10 weeks post infection). Final magnification 100x, 200x and 400x.

Patients with acute schistosomiasis have increased circulating levels of osteopontin in the plasma (p = 0.0005 vs non-infected; p = 0.0005 vs HI and p = 0.0012 vs HS) (Fig 1A) and serum (p = 0.0001) (Fig 1B). The plasma OPN levels in acute schistosomiasis are even higher than in patients with hepatosplenic form of the disease (p = 0.0012) (Fig 1A). We observe that OPN starts to increase in the beginning of the post-postural phase (5–6 weeks post-infection, p = 0.0005 vs non-infected) and OPN levels peaked 7–11 weeks post-infection (p = 0.0001 vs uninfected; p = 0.04 vc 5–6 weeks; p = 0.001 vs 12 weeks and p = 0.0001 vs 24 weeks), when the livers are enriched with necrotic-exudative granulomas (Fig 1C). Twelve weeks after infection the symptoms start to disappear, the granulomas reach a modulated state and circulating OPN levels start to fall, reaching levels comparable to uninfected individuals 24 weeks post-infection (Fig 1C). Receiver operating characteristics (ROC) curve analysis demonstrated that serum OPN measurement could be a good biomarker to identify patients with symptomatic acute schistosomiasis mansoni (Area under the curve = 0.9959; p<0.0001; 95% confidence interval 0.9848–1.007; S1 Fig). In our study population OPN serum test >23.34 can detect a symptomatic acute patient with 95.65% sensitivity and 95.24% specificity (Likelihood ratio = 20.09).

Immunohistochemistry demonstrated that the inflammatory cells in the necrotic-exudative liver granulomas express OPN, especially in the macrophage (epithelioid cells) enriched area around the egg and central necrosis (Fig 1D and S2 Fig).

### Serum and hepatic osteopontin is increased in murine acute schistosomiasis

**Fig 2:**
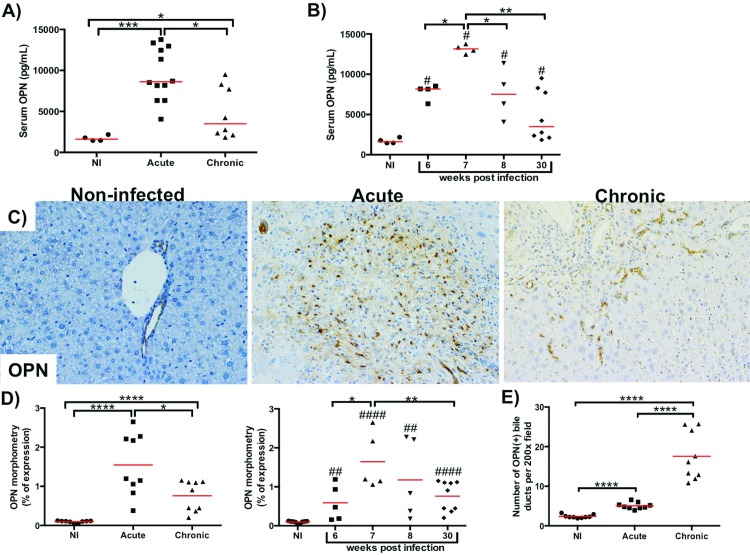
Sera and hepatic osteopontin are upregulated in the acute phase of murine schistosomiasis mansoni. A) ELISA for osteopontin in the serum of uninfected (NI) mice and mice experimentally infected with *S*. *mansoni* in the acute (6,7 and 8 weeks post infection) and chronic phase (30 weeks post infection). Medians are displayed, Mann-Whitney U test: *p<0.05, **p<0.01, ***p<0.001, ****p<0.0001. B) Serum osteopontin ELISA of uninfected controls and infected mice grouped by weeks post infection. Medians are displayed, Mann-Whitney U test: #p<0.05, ##p<0.01, ###p<0.001, ####p<0.0001 vs non-infected; *p<0.05, **p<0.01, ***p<0.001, ****p<0.0001. C) Immunohistochemistry for osteopontin in representative liver section of uninfected mice (left), infected mice in the acute phase (middle) and infected mice in the chronic phase (right). Final magnification 200x. D) Morphometry of the immunohistochemistry for osteopontin. Means are displayed; Student’s t test: #p<0.05, ##p<0.01, ###p<0.001, ####p<0.0001 vs non-infected; *p<0.05, **p<0.01, ***p<0.001, ****p<0.0001. E) Number of osteopontin positive bile ducts per 200x power field. Means are displayed; Student’s t test: *p<0.05, **p<0.01, ***p<0.001, ****p<0.0001.

Similar to humans, mice in the acute phase of infection also have more circulating and hepatic OPN levels than mice in the chronic phase of infection where there is severe fibrosis (p = 0.001 vs Non-infected; p = 0.0124 vs chronic phase) (Fig 2A, 2C and 2D). OPN levels in mice also peaked in the liver (p = 0.0001 vs non-infected; p = 0.0245 vs 6 weeks and p = 0.0104 vs 30 weeks) and serum (p = 0.0286 vs non-infected; p = 0.0286 vs 6 weeks; p = 0.0286 vs 8 weeks and p = 0.004 vs 30 weeks) 7 weeks post-infection, at a time when the livers were enriched with necrotic-exudative granulomas and inflammatory cells (Fig 2B, 2C and 2D). During the acute phase of infection in both mice and humans, the majority of liver OPN producing cells are inflammatory cells (Figs 1D and 2C; S2 Fig), while the ductular reaction is the most important source of OPN in chronic schistosomiasis (Fig 2C and 2E).

### Soluble egg antigens (SEA) induce macrophages osteopontin production

**Fig 3:**
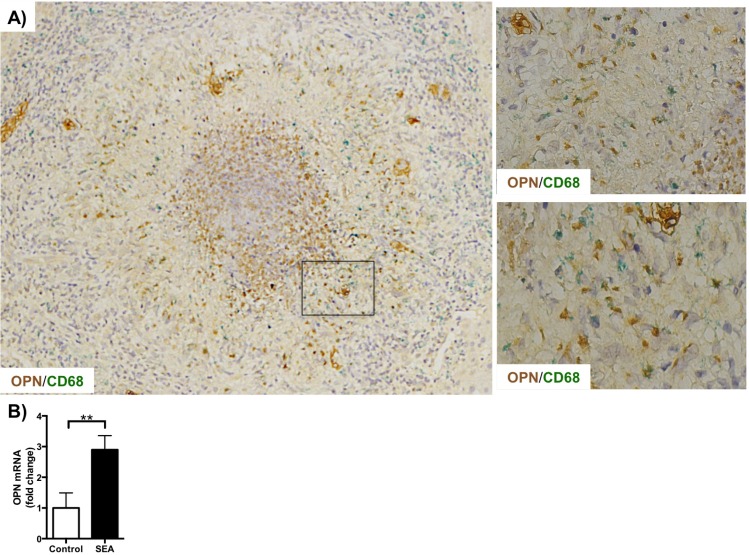
Soluble egg antigens induce Osteopontin expression in primary human Kupffer cells. A) Double Immunohistochemistry for osteopontin (OPN,brown) and CD68 (macrophage marker, green) in liver needle biopsy fragment of a representative subject with acute schistosomiasis mansoni (10 weeks post infection, same case depicted in Fig 1D) demonstrating that macrophages express osteopontin in human acute schistosomiasis mansoni. The rectangle highlights the magnified area shown on the 2 pannels on the right. Final magnification 100x (left), 400x (upper right) and 600x (lower right). B) Osteopontin mRNA expression (fold change) in primary human Kupffer cells incubated with 10 μg/ml SEA or 0.0001 μg/ml LPS (lipopolysaccharide; control, same amount of endotoxin present in the SEA preparation) after 3 hours of incubation. Mean±SEM are displayed, Student’s t test: *p<0.05, **p<0.01, ***p<0.001, ****p<0.0001.

OPN expression in both human and murine acute schistosomiasis is enriched in the macrophage area of the necrotic-exudative granulomas. Double immunohistochemistry for OPN and CD68 (a macrophage marker) confirmed that the macrophages in acute schistosomiasis express this pro-inflammatory cytokine (Fig 3A). Since the macrophages are in contact with egg antigens, we investigated if soluble egg antigens could stimulate OPN production *in vitro*. Primary human Kupffer cells incubated with SEA for 3 hours upregulated OPN mRNA (p = 0.0082) (Fig 3B), indicating that infection *per se* can directly increase macrophage expression of this proinflammatory and profibrogenic molecule.

## Discussion

We demonstrated for the first time that circulating osteopontin levels are increased in human acute schistosomiasis mansoni. Our results also suggest that serum OPN measurement could be a good biomarker to diagnose symptomatic acute schistosomiasis. The highest levels of OPN were observed in patients during the peak of clinical symptoms (7–11 weeks post infection). Once the granulomas were modulated (>12 weeks post infection) the OPN levels decrease significantly.

Circulating and hepatic OPN levels were also elevated in the acute phase of experimental murine schistosomiasis mansoni. Chen et al. (2011) demonstrated that liver OPN levels peaked at the acute phase of *S*. *japonicum* infection. As previously mentioned, the murine model has some limitations in regard to acute schistosomiasis [14]. However the model may be helpful to identify the factors related to the onset of the generalized reactive changes during the early course of a primary schistosomal infection [14]. Importantly, our new data in humans demonstrate that the mouse model mirrored the human disease with regards to the pattern of OPN expression, reinforcing that this model could be useful to understand the mechanisms related to the acute phase of schistosomiasis in humans.

Macrophages are the major OPN producing cell in acute schistosomiasis and SEA induces OPN expression in primary human Kupffer cells. Pereira et al. (2015) also observed that macrophages are one of the major sources of OPN in the early phases of infection in mice and in patients with hepatointestinal schistosomiasis, while bile ducts are the main producers of OPN in patients with hepatosplenic disease. We confirm that osteopontin is mostly expressed by the ductular reaction in mice in the late chronic phase of infection. Pereira et al. (2015) also observed that SEA stimulates primary mouse Kupffer cells, stellate cells and cholangiocytes to produce OPN, demonstrating that egg antigens directly induce the expression of this pro-inflammatory and pro-fibrogenic molecule by multiple types of cells that localize in schistosoma-infected livers.

Osteopontin has been previously associated with acute hepatic injury [16, 17, 22, 23]. Patients with acute liver failure of different etiologies such as acetominophen toxicity, ischemia (shock), idiosyncratic drug-induced liver injury, autoimmune hepatitis and viral hepatitis A and B, have increased OPN plasma levels [22, 23]. Recent findings indicate that OPN plays a central role in liver diseases associated with necrosis [16, 17, 23]. Liver injury triggers OPN production in Kupffer cells and NKT cells that attract neutrophils, lymphocytes and macrophages to affected areas [16, 17, 19, 24]. The recruited cells become activated and produce OPN and Th1 cytokines, exacerbating liver necrosis [16, 17, 19, 24]. In acute liver failure patients, OPN was particularly associated with hyperactute injury [23].

The role of OPN has been described in granulomatous reactions, especially Th1-mediated, [16, 19]. OPN is essential for Th1 polarization [25] and OPN from dendritic cells mediates granuloma formation against bacterial antigens [26]. OPN expression in sarcoidosis, tuberculosis and other Th1-mediated granulomas is more associated with macrophages than extracellular matrix [27]. Using the B-glucan model, Morimoto et al. (2004) demonstrated that OPN-/- mice have a reduction in granuloma size and number and a 2-fold decrease in macrophage accumulation [28]. Overexpression of OPN increased granuloma formation and delayed its resolution, promoting an exacerbated fibrotic response [28]. Similar findings were observed by O’Regan and coworkers (2008) in *S*. *mansoni* egg-induced lung granulomas, a typical Th2-mediated granuloma [20]. Our results confirm the pivotal role of OPN in the Th1 and Th2 mediated granulomas and demonstrate that pathogen antigens directly induce OPN production by macrophages.

Acute schistosomiasis is a systemic hypersensitivity reaction against *S*. *mansoni* and it is characterized by miliary distribution of hyperergic necrotic-exudative granulomas [2]. The live miracidia inside the egg secrete a series of antigens and lytic substances that can trigger OPN production, recruiting inflammatory cells and inducing the granulomatous reaction to prevent further liver damage (Th1 over Th2 response) [2, 3, 10, 18]. As disease progress (Th2 over Th1 response), the granulomas are modulated (decrease in IFN-gamma and increase in IL10), the antigens and lytic substances are sequestered, necrosis is no longer observed and OPN is down regulated [2, 3, 10, 12]. Patients that will develop hepatosplenic schistosomiasis continue to produce OPN, especially by the ductular reaction, promoting fibrosis and portal hypertension [18].

The plasma levels in acute schistosomiasis were even higher than observed in hepatosplenic patients. Although OPN was demonstrated to be stable in both serum and plasma, OPN levels in the serum are 3.8–4.8 times lower than in plasma [29]. The authors speculate that this phenomenon may reflect OPN sequestration by the clot or its cleavage by thrombin, leading to loss of immunoreactivity [29]. In our cohort of acute patients only a small number of individuals had both plasma and serum samples collected and we also observed a 4–4.5 times reduction of OPN levels in serum compared to plasma (S1 Table). Ideally, future studies should use plasma samples in order to measure the total amount of circulating osteopontin.

In conclusion, *S*. *mansoni* egg antigens induce the production of OPN by macrophages in the necrotic-exudative granulomas characteristic of acute schistosomiasis mansoni. Circulating OPN levels are upregulated in human and murine acute schistosomiasis and could be a non-invasive biomarker of this form of disease.

## Supporting Information

S1 Checklist STROBE Checklist. (PDF)

S1 Fig Osteopontin is a good biomarker to identify patients with symptomatic acute schistosomiasis mansoni. (PDF)

S2 Fig Epithelioid cells in the periovular granuloma express the pro-inflammatory and pro-fibrogenic molecule osteopontin. (PDF)

S1 Table Serum and Plasma osteopontin levels from two patients with acute schistosomiasis mansoni. (PDF)

S1 Dataset Dataset of all variables used in the statistical analysis (XLS)
