# Supplementary material for: Osteopontin Is Upregulated in Human and Murine Acute Schistosomiasis Mansoni
Source: PLoS Negl Trop Dis. 2016 Oct 18;10(10):e0005057. doi: 10.1371/journal.pntd.0005057 (PMC5068698; doi:10.1371/journal.pntd.0005057)
Supplement: S1 Fig — (PDF) [file pntd.0005057.s002.pdf]

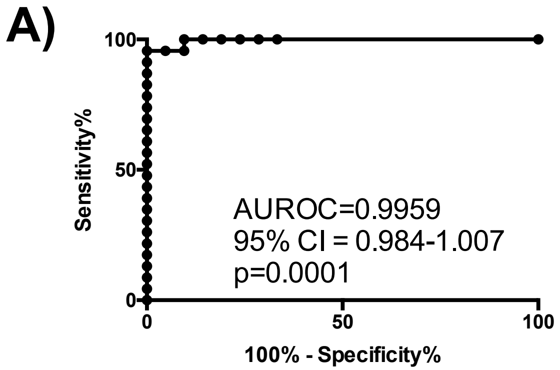

A)
100
50
0
Sensitivity%
AUROC=0.9959
95% CI = 0.984-1.007
p=0.0001
0
50
100
100% - Specificity%

**S1 Fig – Osteopontin is a good biomarker to identify patients with symptomatic acute schistosomiasis mansoni. A)** Receiver operating characteristics (ROC) curve analysis demonstrating that serum OPN measurement could be a good biomarker to identify patients with symptomatic acute schistosomiasis mansoni (Area under the curve (AUROC) = 0.9959; $p<0.0001$; 95% confidence interval 0.9848-1.007).
