# Supplementary material for: Osteopontin Is Upregulated in Human and Murine Acute Schistosomiasis Mansoni
Source: PLoS Negl Trop Dis. 2016 Oct 18;10(10):e0005057. doi: 10.1371/journal.pntd.0005057 (PMC5068698; doi:10.1371/journal.pntd.0005057)
Supplement: S2 Fig — (PDF) [file pntd.0005057.s003.pdf]

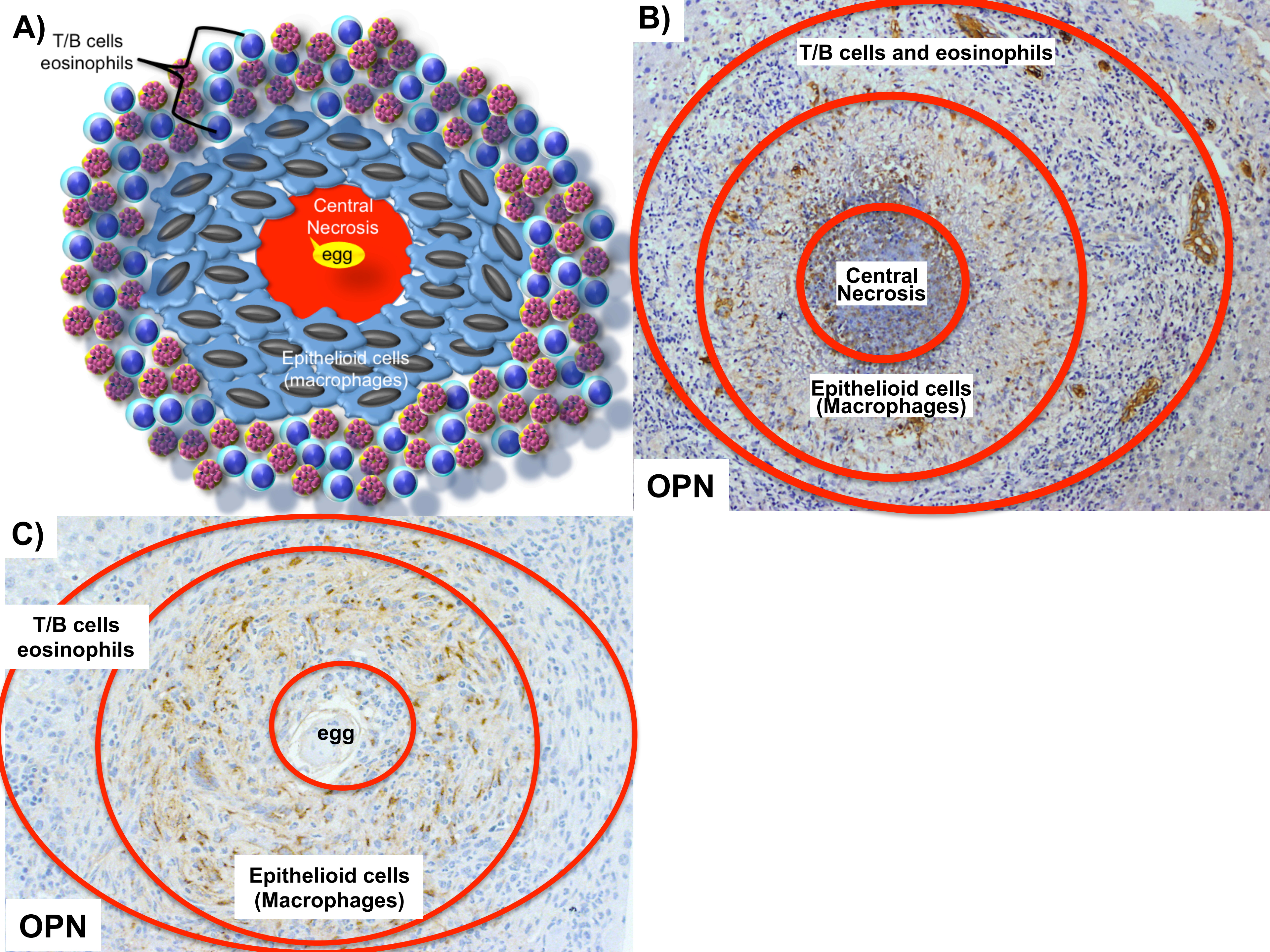

A)
T/B cells
eosinophils
Central
Necrosis
egg
Epithelioid cells
(macrophages)
B)
T/B cells and eosinophils
Central
Necrosis
Epithelioid cells
(Macrophages)
OPN
C)
T/B cells
eosinophils
egg
Epithelioid cells
(Macrophages)
OPN

S2 Fig – Epithelioid cells in the periovular granuloma express the pro-inflammatory and pro-fibrogenic molecule osteopontin. A) Schematic representation of the granulomatous reaction against Schistosoma mansoni eggs during the acute phase of infection. Note the characteristic central necrosis surrounding the egg, followed by a zone of epithelioid cells (macrophages) and imediatelly after that a zone enriched with CD4+ and CD8+ T cells, B cells and eosinophils. B) Immunohistochemistry for osteopontin in liver needle biopsy fragment of a representative subject with acute schistosomiasis mansoni (10weeks post infection). Observe that the zone of epithelioid cells in the granuloma has the most immunoreactivity for osteopontin. Final magnification 100x. C) Immunohistochemistry for osteopontin in liver fragment of a representative infected mice with acute schistosomiasis mansoni (7 weeks post infection). Note that the central necrosis is not as evident as in human acute schistosomiasis mansoni. Final magnification 200x.
