# Supplementary material for: Osteopontin Is Upregulated in Human and Murine Acute Schistosomiasis Mansoni
Source: PLoS Negl Trop Dis. 2016 Oct 18;10(10):e0005057. doi: 10.1371/journal.pntd.0005057 (PMC5068698; doi:10.1371/journal.pntd.0005057)
Supplement: S1 Table — (PDF) [file pntd.0005057.s004.pdf]

S1 Table – Serum and Plasma osteopontin levels from two patients with acute schistosomiasis mansoni. Plasma and serum samples were collected on the same day and the samples were run in the same ELISA assay plate.

Patient	Sex	Age	Plasma OPN	Serum OPN	Fold change
20	male	16	417.61 ng/mL	90.92 ng/mL	4.59
21	male	66	367.49 ng/mL	89.87 ng/mL	4.08
